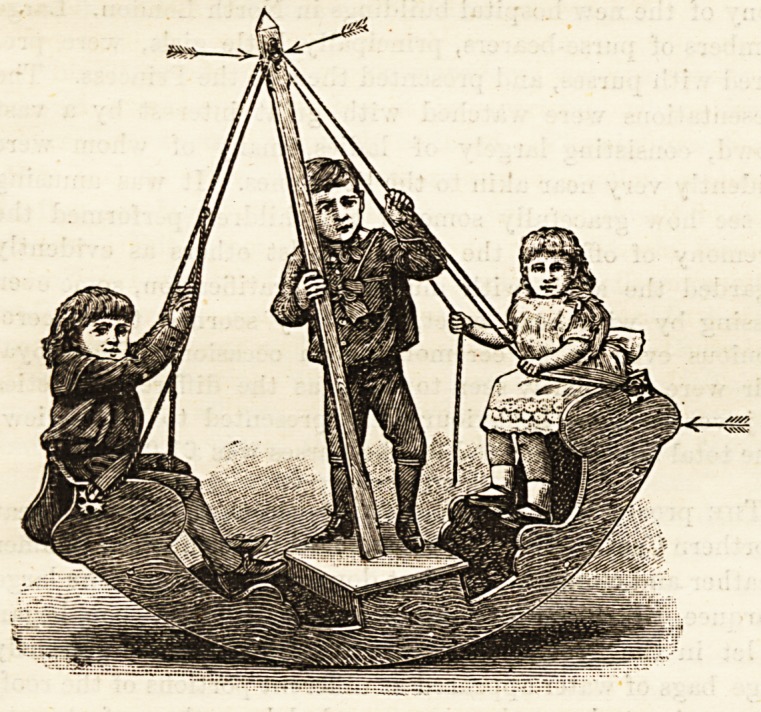# New Remedies and Appliances

**Published:** 1888-07-21

**Authors:** 


### NEW REMEDIES AND APPLIANCES.

SANDELL'S CASCARA TABLETS.—Mr. Sandell has prepared these tablets with the object of minimising the peculiar taste which makes the fluid preparations of cascara so unpleasant. They consist of an intimate mixture of chocolate with cascara extract. We have given them several trials. They are exceedingly pleasant to take, and possess all the mild laxative properties of the liquid cascara. Those who object to pills, powders, and mixtures will probably find the tablets much to their taste. For children especially they will be found very convenient.

THE NEW NURSERY BOAT OR YACHT.—Dr. C. R. Illingworth has invented and patented a nursery boat or yacht, which is not only novel, but will prove a decided boon to all who have many children whom they find it difficult to amuse. We give herewith a sketch of the boat, which shows its distinctive features. It is broad in the middle, and cannot topple laterally; it is made with blocked ends, which completely prevent its tilting. The boat is worked with ropes and pullies, which the little ones themselves lay hold of and pull, thus securing exercise for the chest and arms whilst amusing themselves. It is in every respect a decided improvement on the old rocking-horse. Half-a-dozen children can use it at once, and as the little ones are strapped in, the whole nursery may join at the same game with absolute safety.

WIESBADEN KOCHBRUNNEN SALTS.—These salts are strongly recommended in the various catarrhs which are so trying to many people of middle and advanced life. For catarrh of throat or chest, of stomach or of bowel, they are said to be prompt and efficacious in action. They are very mild, and may be taken for weeks in succession with advantage. A teaspoonful daily dissolved in water acts as a mild aperient. During the recent severe weather, when throat and chest catarrhs have been unusually prevalent, we have made several trials of the salts, and always with satisfactory results. They are liked by patients, and so far as a limited experience shows, deserve the reputation they have already won.

WIESBADEN KOCHBRUNNEN PASTILLES.—The pastille is an excellent form in which to take remedies of this kind. Pastilles made of similar salts were some time ago strongly recommended by Sir Morell Mackenzie for certain throat affections. The Kochbrunnen pastilles are really pleasant sweets, and are eaten freely with great satisfaction by those who like ordinary sweets. Delicate ladies and children, and all who object to nauseous mixtures, should keep a few boxes by them in damp weather. In all stages of a cold, or of throat or chest affections, they are of decided service.

WIESBADEN KOCHBRUNNEN WATER.—This water contains the Kochbrunnen salts in solution. It is used for the same purpose as the salts and pastilles, viz., in catarrhs of all kinds. In the common derangements of stomach and bowel it is very serviceable. Its mildness is much in its favour. The most delicate may take it freely without fear. It is usually taken warm, and for this purpose is placed in a vessel of hot water, and raised to the temperature desired. A daily dose, taken either hot or cold, for a month, will be found of great service by those who are troubled with inactive bowels or a sluggish liver.

WIESBADEN KOCHBRUNNEN SOAP.—A soap has been prepared containing a certain proportion of the Kochbrunnen salts. As a soap it is most pleasant, and in moist or eczematous conditions of the skin is exceedingly valuable. All these preparations are sold by Messrs. Ingram and Royle, of Farringdon Street, whose reputation in all that appertains to mineral waters is world-wide, and an absolute guarantee of genuineness.

## Figures and Tables

**Figure f1:**